# The margin of internal exposure (MOIE) concept for dermal risk assessment based on oral toxicity data – A case study with caffeine

**DOI:** 10.1016/j.tox.2017.03.012

**Published:** 2017-12-01

**Authors:** Jos G.M. Bessems, Alicia Paini, Monika Gajewska, Andrew Worth

**Affiliations:** Directorate Health, Consumers and Reference Materials, European Commission, Joint Research Centre, Ispra, Italy

**Keywords:** AUC, area under the curve, BMDL, benchmark dose lower confidence interval, C_max_, maximum concentration, HLV, human limit value, MOE, margin of exposure (synonymous to MOS), MOIE, margin of internal exposure, MOS, margin of safety, NOAEL, no observed adverse effect level, PBK, physiologically based kinetic, POD, point of departure, RCR, risk characterisation ratio, SED, systemic exposure dose, Risk assessment, Route-to-route extrapolation, Physiologically based kinetic (PBK) model, Margin of internal exposure (MOIE), Caffeine, Physiologically based pharmacokinetic (PBPK) model

## Abstract

Route-to-route extrapolation is a common part of human risk assessment. Data from oral animal toxicity studies are commonly used to assess the safety of various but specific human dermal exposure scenarios. Using theoretical examples of various user scenarios, it was concluded that delineation of a generally applicable human dermal limit value is not a practicable approach, due to the wide variety of possible human exposure scenarios, including its consequences for internal exposure. This paper uses physiologically based kinetic (PBK) modelling approaches to predict animal as well as human internal exposure dose metrics and for the first time, introduces the concept of Margin of Internal Exposure (MOIE) based on these internal dose metrics. Caffeine was chosen to illustrate this approach. It is a substance that is often found in cosmetics and for which oral repeated dose toxicity data were available. A rat PBK model was constructed in order to convert the oral NOAEL to rat internal exposure dose metrics, i.e. the area under the curve (AUC) and the maximum concentration (C_max_), both in plasma. A human oral PBK model was constructed and calibrated using human volunteer data and adapted to accommodate dermal absorption following human dermal exposure. Use of the MOIE approach based on internal dose metrics predictions provides excellent opportunities to investigate the consequences of variations in human dermal exposure scenarios. It can accommodate within-day variation in plasma concentrations and is scientifically more robust than assuming just an exposure in mg/kg bw/day.

## Highlights

• The concept of Margin of Internal Exposure (MOIE) is proposed for route-to-route extrapolation.
• It is an extension of the Margin of Exposure (MOE) approach for cosmetics in the EU.
• PBK modelling integrates *in vitro* and *in silico* predictions of ADME-properties.
• The MOIE approach is transparent and facilitates to make uncertainties explicit.
• The MOIE can be extended to include *in vitro* toxicity data in animal-free risk assessment.

## 1 Introduction

The human health risk of exposure to a chemical can be characterized in two ways. The first approach is to use a risk characterisation ratio (RCR) approach where the human exposure level is compared to a pre-established human limit value (HLV), usually based on an animal point of departure (POD) divided by relevant assessment factors. The second approach, the margin of exposure (MOE) approach, is synonymous to the margin of safety (MOS) approach and is used in the EU for the safety evaluation of cosmetic products and ingredients as long as they are still based on animal toxicity data that were generated before the 2013 ban on animal testing (SCCS, 2016). In the MOE approach the human exposure is directly compared to a POD (BMDL or NOAEL) from an oral bioassay. Although performed on a case-by-case basis, usually a value of 100 is used, a factor 10 for interspecies extrapolation and another factor 10 to account for inter-individual variations. These factors can be further subdivided and where appropriate, replaced or amended by chemical-specific factors. For example, incomplete human absorption is taken into account when expressing the MOE between the BMDL (or NOAEL) and the human systemic exposure dose (SED):

$$\text{MOE}=\frac{\text{BMDL}/\text{NOAEL}}{\text{SED}}$$

The SED is based on external exposure estimates and route-relevant absorption figures. The case-by-case MOE approach also allows taking into account the vast amount of widely varying human exposure scenarios that can even encompass more than one route of exposure for the same person at the same time. The intended application of cosmetics obviously is always dermal (topical application), often with unintentional inhalation exposure (e.g. aerosol formation) or even unintentional oral exposure (e.g. toothpaste). The number of use frequencies, the amounts applied and the skin areas covered are indicated in Table 1 in the SCCS Notes for Guidance (SCCS, 2016). Furthermore, one ingredient may appear in different cosmetic product categories. One ingredient (e.g. a preservative) might be used in eye liner where skin surface area exposed is very small (3.2 cm^2^), face cream (565 cm^2^, equal to ½ head female area), in hand cream (860 cm^2^, equal to hands area) and in sunscreen (17500 cm^2^, equal to total body area). As a consequence, the same concentration of an ingredient, but each time in a different product type, may result in different systemic exposure patterns and levels. Using somewhat rounded skin surface area (SSA) numbers for simplification, this is illustrated theoretically in Table 1, where the maximum flux (J_max_ = P_app_ * SSA * C in μg/h) is used as an approximation of the rate at which an ingredient may penetrate the exposed skin. In the second last column, of Table 1, the total time (t, in h) that would take to absorb (from the selected part of the body) the total mass of the ingredient in the cosmetic product that was applied is reported. For eyeliner, it takes 50 h to absorb only 0.5 μg, for face cream, it takes only 0.5 h to absorb 5 μg and for sunscreen, it takes only 1 h to absorb 100 μg. Assuming that the elimination (metabolism + excretion) of the ingredient is slow (absorption-limited kinetics), one can imagine that using the same concentration of an ingredient (e.g. a product limit) in an eyeliner will result in low steady state plasma concentration, where in a face cream an amount ten times more is causing a very sharp peak of only 0.5 h. Lastly, with use in sunscreen, two hundred times more mass is absorbed in just 1 h. In other words, the mass absorbed per hour is not a percentage that is a constant for various products. It is very much dependent on the surface area dose that is 50 times lower for sunscreen as compared to eyeliner in the theoretical example. Larger exposed skin areas go with lower surface area dose, but higher body burden as of the high surface area, i.e. higher relative absorption. This showcases the scientific limitations of using relative dermal absorption as a constant for the many types of cosmetic products as well as the widely differing surface area dose.

**Table 1:** Theoretical example of three use scenarios of a cosmetic product containing the same concentration of a cosmetic ingredient.^a^

J_max_ = P_app_ * SSA * C	Concentration in product	Volume applied	Amount applied	Exposed skin area	Surface area dose	App. perm. coefficient	Max. body load per h	Duration for total absorption	External exposure (50 kg)
	C			SSA	SAD	P_app_	J_max_	t	
	μg/cm^3^	cm^3^	μg	cm^2^	μg/cm^2^	cm/h	μg/h	h	μg/kg bw/d
Eyeliner	5	0.1	0.5	2	0.25	0.001	0.01	50	0.01
*factor*	*=*	*10*	*10*	*250*	*0.04*	*=*	*250*	*0.01*	*10*
Face cream	5	1	5	500	0.01	0.001	2.5	0.5	0.1
*factor*	*=*	*20*	*20*	*40*	*0.5*	*=*	*40*	*2*	*20*
Sunscreen	5	20	100	20000	0.005	0.001	100	1	2

a Large differences in the total skin exposure area could result in completely different body loads per hour (J_max_) and concentration-time profiles as indicated by duration for total absorption. Assuming that plasma clearance at least for face cream and sunscreen scenarios is smaller than total dermal penetration (J_max_), i.e. if the sum of metabolism and excretion are rate-limiting and absorption is not balanced by total clearance, the C_max_ values could differ significantly quite well.

The EU SCCS provides guidance on how to establish dermal absorption values (SCCS, 2012, SCCS, 2016): if no other data is available, it is advised to use 50% as a default number. The preferred option however is to study the dermal absorption *in vitro* according to the OECD TG 428 (*in vitro* dermal absorption) and under relevant exposure conditions. Unfortunately, as mentioned by the SCCS Notes for Guidance (Chapter 3–4.1.1 Dermal/percutaneous absorption; SCCS, 2016), *in vitro* measurements using less than 2 mg/cm^2^ skin surface area dose (SSAD) are not technically feasible while the amounts of cosmetic products applied to skin usually do not exceed an SSAD of 1 mg/cm^2^ under *in use* conditions. If indicated, the default systemic exposure for the oral route in the animal bioassay (100%) to be used in the formula for route-to-route extrapolation can be amended to 50% or 10%.

A shortcoming in both approaches is the apparent lack of attention for species- and route-dependent metabolism. Especially, the first pass metabolism when the test substance passes the liver in an animal oral bioassay may differ significantly from the first pass metabolism in skin upon human exposure, qualitatively as well as quantitatively. Metabolites, if formed at all, may differ and the rate of formation of the (various) metabolites may differ as well. In summary, route-to-route (including interspecies) extrapolation is not easy to apply since it is associated with considerable uncertainties, including the difficulty of accounting for the occurrence of local effects, the rate of absorption and possible ‘first pass’ metabolism during absorption (Pepelko and Withey, 1985, Pepelko, 1987, Rennen et al., 2004).

**Fig. 1:**
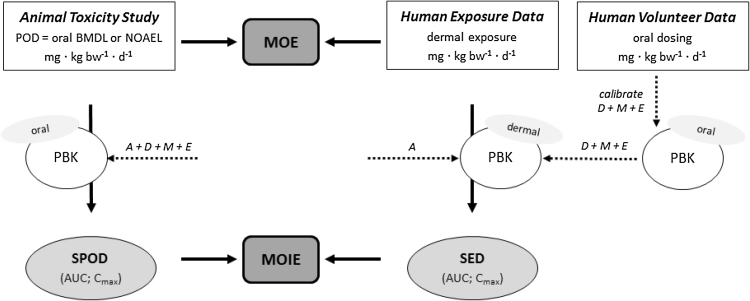
Conceptual depiction of the margin of internal exposure (MOIE) approach based on comparison of internal dose metrics. The individual assessment factor (AF) of 4 that should cover interspecies differences in toxicokinetics (TK) can be left out in the MOIE approach as these differences are taking into account using the PBK approach (animal PBK model and human PBK model). aPBK = animal PBK model; hPBK = human PBK model. SED = Systemic exposure dose; SPOD = Systemic Point of Departure.

To overcome the lack of experimental data, a computational approach seems the best approach to apply. This is critical to be able to take into account the relevant uncertainties that accompany the various extrapolations needed (interspecies, route-to-route, high-to-low dose and scenario-to-scenario). Physiologically based kinetic (PBK^1^) models, can be used to reduce these uncertainties, they can be used to simulate animal and human concentration-time profiles of a parent chemical and its (relevant) metabolites in the blood and organs following a specific exposure scenario (Paini et al., 2012, Louisse et al., 2012, Punt et al., 2016). They can accommodate various sorts of input data, be they determined *in vitro* or predicted *in silico* via QSARs (Bessems et al., 2014). Once sufficiently evaluated using experimental data, they can be useful in comparing systemic exposure dose metrics between species and routes of exposure in a modified MOE approach presented for the first time in this paper: the margin of internal exposure (MOIE) approach (Fig. 1). Examples of internal dose metrics are C_max_ (the maximum concentration simulated in blood plasma) and AUC (the simulated area under *the concentration-time* curve) following oral, dermal and inhalation exposure in the species of interest. In addition, aggregate co-exposure via multiple routes and from multiple cosmetic products may be taken into account.

**Fig. 2:**
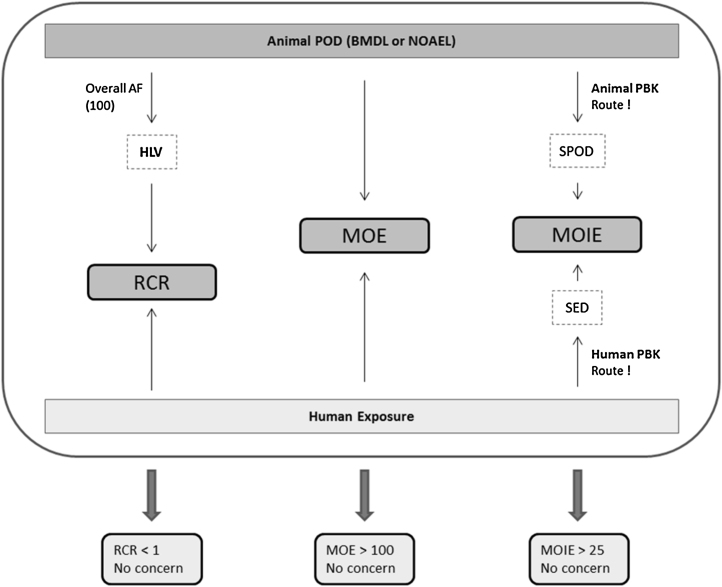
Two options to take assessment factors (AFs) into account in the risk characterisation process. Option 1 is the a priori use of AFs to establish a ‘Human Limit Value’ based on the Hazard Characterisation. Option 2 establishes whether the Margin of Exposure (MOE) between the exposure and the POD is larger than the overall assessment factor (being the product of the individual AFs or a probability distribution of the product of the distributions of the individual AFs. The result of option 1 is a Risk Characterisation Ratio (RCR). RCR < 1 means risk is under control. The result of option 2 is an MOE being smaller or larger than the overall AF (concern/no concern).

The present paper presents a methodological case study following the MOIE approach to characterise the risk of caffeine exposure in a dermal product using data based on an oral bioassay (Fig. 2). Caffeine was chosen for this case study for oral-to-dermal extrapolation since it is an ingredient in cosmetics and oral animal toxicity data are present. A rat PBK model was developed for caffeine to convert the chosen oral NOAEL to the internal dose metrics AUC and C_max_ of caffeine, as well as of its most relevant metabolite paraxanthine. Secondly, we used an oral human PBK model (Gajewska et al., 2014, Gajewska et al., 2015) to predict human internal dose metrics. Data from an oral human volunteer study were used to calibrate some of the PBK model parameters. Subsequently, the calibrated human oral model was extended with a dermal compartment to accommodate a few realistic exposure scenarios involving the topical use of cosmetic products. QSARs were used to simulate human dermal penetration. Finally, the resulting internal dose metrics for the rat (oral) and the human (dermal) were compared in terms of resulting MOIE. With the present work we cover the extrapolations oral to dermal, high rat to low human as well as single to repeated.

## 2 Approach

### 2.1 Caffeine

**Fig. 3:**
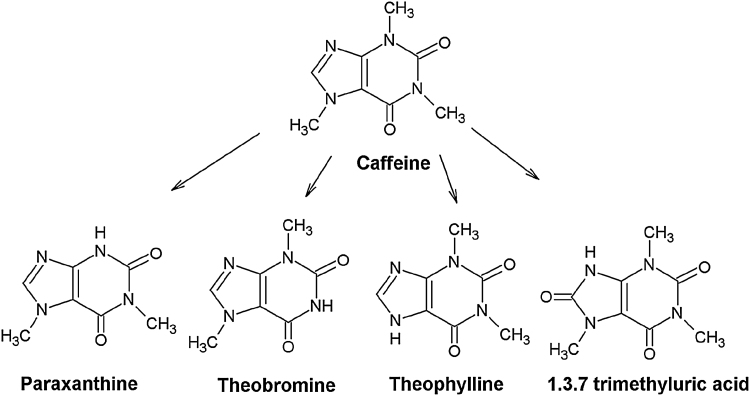
Caffeine and major metabolites formed in the liver.

The alkaloid caffeine has potent antioxidant properties and is used in many creams and lotions since it is believed to slow down the photo aging process of the skin by absorbing ultraviolet radiation (skin cancer prevention). It is also present as active compound in anti-cellulite products (claim to prevent excessive accumulation of fat in cells). It supposedly increases the microcirculation of blood in the skin and stimulates the growth of hair through inhibition of the 5-α-reductase activity (Herman and Herman, 2012 and references therein). The toxicokinetics of caffeine including its metabolism and the use of this information in PBK modelling have been described in previous studies (OECD SIDS, 2002; Csajka, Haller, Benowitz, & Verotta, 2005; Ginsberg, Hattis, Russ, & Sonawane, 2004; Lelo, Birkett, Robson, & Miners, 1986; Zandvliet et al., 2005, Thorn et al., 2012, Gajewska et al., 2014, Gajewska et al., 2015). Caffeine is rapidly absorbed from the gastrointestinal (GI) tract and almost completely metabolized (≤3% excreted unchanged in urine). The four primary metabolic conversions in humans that are taken into account in this case-study PBK modelling approach are N-3 demethylation to paraxanthine, N-1 demethylation to theobromine, N‐7 demethylation to theophylline and C-8 hydroxylation to form 1,3,7-trimethyluric acid (Fig. 3). They account for approximately 84%, 12%, 4% and less than 1%, of total caffeine metabolism, respectively.

### 2.2 Selected animal oral POD

The current case study approach is not intended to be an in-depth risk assessment. The choice of the oral POD was taken from an OECD assessment (OECD SIDS, 2002). As the purpose of the current case-study is not to scrutinise existing information on hazard characteristics, we have arbitrarily chosen 10 mg/kg bw/d as oral POD, being the most conservative POD found in the OECD SIDS report. It is the lowest NOAEL mentioned, it’s based on fetotoxicity at the LOAEL following administration via the drinking water in a rat developmental toxicity/teratogenicity study. In the same OECD SIDS report from a similar study, but now with gavage administration, maternal toxicity is reported at the lowest dose level tested (LOAEL 10 mg/kg bw/d; NOAEL <10 mg/kg bw/d). As far as we are aware, no clarity exists as to the supposed mode of action (MOA) underlying the NOAEL and LOAEL mentioned above, be it caffeine, (a) metabolite(s), or a combination of parent compound and metabolites.

### 2.3 Dose metric

As no solid information was available on the MOA, the PBK modelling and the establishment of the MOIE included both caffeine as well as the most relevant metabolite (paraxanthine) in terms of relative level of formation.

### 2.4 Selected human exposure scenarios

In order to calibrate some model parameters in the human PBK model, data from a human volunteer study (caffeine in a gelatine capsule ingestion) were used (for more information see Gajewska et al., 2015).

For the establishment of the MOIE, a scenario assuming the skin application of a sun screen was selected, being equivalent to 2.5 mg/kg bw of caffeine. The value of 2.5 mg/kg bw was chosen for the human dermal scenario as to avoid the risk of linearity versus non-linearity of the ADME processes (absorption, distribution, metabolism and excretion) between the oral rat and dermal human simulations. See Table 2 for an overview of rat and human exposure/dose levels.

**Table 2:** Selected rat (oral) and human (oral and dermal) exposure conditions for caffeine including the classical MOE.

Rat oral	
POD [mg/kg bw]	10.1

Human oral calibration	
Dose [mg/kg bw]	2.5
Body weight [kg]	65
Dose [mg]	162.5
Simulation time [h]	24
Administration type	Gelatine tablet
Dosing	Repeated (4 times, every 2 h)

Human dermal	
Dose [mg/kg bw]	2.5
Body weight [kg]	75
Dose [mg]	187.5
Skin area [cm^2^]	500 (rounded from 565)^a^
Skin dose (skin loading, skin surface area dose) [mg/cm^2^]	0.375
Exposure time [h]	6
Dosing	Single and Repeated (4 times, every 2 h)
Simulation time [h]	100
Vehicle	Ethanol + propylene glycol
Concentration of formulation [mg/mL]	4.56
Occlusion	Vehicle evaporation up to 8 h
MOE (Margin of Exposure) = (Rat external POD)/(Human External Dose) (10.1 mg/kg bw)/(2.5 mg/kg bw)	**4**

a 565 cm^2^ is in Table 1 of the SCCS Notes of Guidance (2016) for face cream.

### 2.5 PBK model structure, assumptions and parameterisation

**Fig. 4:**
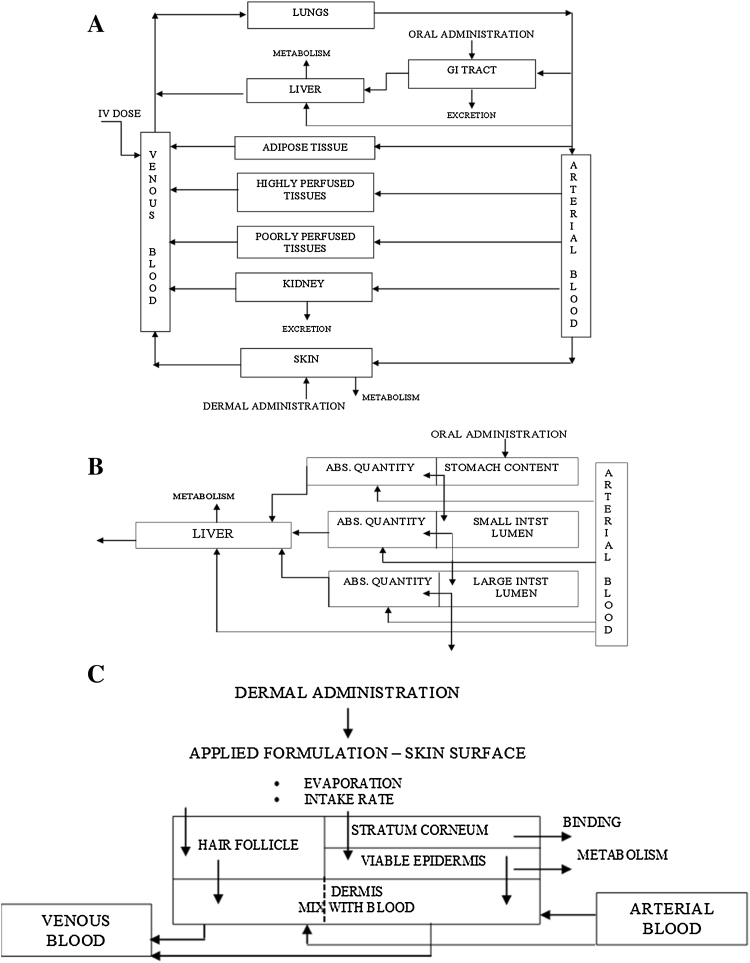
a. General structure of the PBK model. b. Sub-compartments of the GI tract, which here is divided into sub-compartments in the human PBK model. c. Skin divided into sub-compartments in human PBK model for dermal exposure.

We illustrate the integrated use of different modelling approaches to simulate the *in vivo* kinetics of dermally applied caffeine. At the heart of this are a series of PBK models that were developed here or earlier: an oral rat model and a dermal human model for which some elimination parameters were calibrated using oral human volunteer data and the dermal absorption by using dermal human volunteer data. QSAR modelling was used to predict dermal absorption information needed for the human dermal PBK model. The various theoretical models were applied and integrated in order to develop an approach for assessing dermal bioavailability. Parts of the human PBK model were published before (Gajewska et al., 2014, Gajewska et al., 2015). The dermal human model was based on the human oral PBK model (Gajewska et al., 2014). The oral rat model was developed for the current paper by replacing human parameters with rat parameters as far as rat specific (rat physiology and anatomy, see appendix). Chemical specific parameters were kept the same as in the human model which had been set after optimization to the human volunteer blood data (Gajewska et al., 2014). See Fig. 4 for the overall PBK model structures.

The PBK modelling approach was based on the following assumptions:

a) In the human models, skin and GI tract are represented by sub-compartments, Fig. 4b and c. The sub-compartments account for the complexity of the absorption process (especially the time-lag in absorption). In the rat PBK model the GI tract is represented by a single compartment and a 1st order rate absorption is assumed.
b) In the human PBK model for oral absorption, the GI tract is represented by 6 sub-compartments (Fig. 4b). In modelling absorption along the GI tract following gavage administration dissolution from matrix, stomach emptying rate and a first order rate of absorption from stomach, small and large intestine were taken into account.
c) In the rat PBK model for oral absorption, the GI tract is represented by a single compartment with first order rate of absorption following administration via food or water for caffeine (light meal followed directly by intake of a little water in which the caffeine was dissolved which means that exposure of the stomach is actually represented as food exposure). The value of this absorption rate and of metabolic parameters (liver metabolism) were obtained by fitting to oral rat experimental data for caffeine (Mohiuddin et al., 2009).
d) For PBK modelling of human dermal exposure, 4 compartments were used for dermal exposure (surface compartment for the product formulation and 3 skin sub-compartments: *stratum corneum*, viable epidermis and dermis perfused by blood), with the addition of one extra skin sub-compartment representing hair follicles (see Fig. 4c). This model was selected because it gave the best goodness of fit (Gajewska et al., 2014).
e) Unidirectional diffusion describes the one-way transport in fine skin (skin without hair follicles) and hair follicles according to Fick’s second law with specified initial and boundary conditions. The diffusion coefficients are different for the *stratum corneum*, viable epidermis and hair follicles and assumed constant throughout the skin absorption process. It is assumed that the test compound is applied onto the skin surface in a pure solvent (vehicle) to account for a simple formulation (i.e. in ethanol, acetone). No mixture effects are considered and the vehicle is assumed to disappear from the skin surface (in model constructed as separate compartment) only due to possible evaporation.
f) Metabolism is assumed to occur only in the liver.
g) The liver metabolism was assumed to follow Michaelis–Menten kinetics. The V_max_ and K_m_ used in the present work are reported in Gajewska et al., 2014.
h) Excretion via urine is described by a first order rate constant.
i) Tissues are assumed to be homogenised compartments with respect to the concentration of a chemical (instantaneous distribution of chemical or metabolite once it is delivered by the arterial blood). Transport between blood and tissues is assumed to be flow-limited (assuming that transport barriers between free molecules of chemical in blood and tissue are negligible) and equilibrium between free and bound fractions in blood and tissue is instantaneous. We used the QSAR-based Schmitt approach to calculate a partition between the blood and the organs of interest (Schmitt, 2008). The model calculates steady-state tissue:plasma partition coefficients based on the composition of the tissues in terms of water, neutral lipids, neutral and acidic phospholipids and proteins using the lipophilicity, the binding to phospholipid membranes, the pKa and the unbound fraction in blood plasma as compound specific parameters. For caffeine, calculations were done for pKa 10.4, LogP_oct_ = −0.07 (octanol-water partition coefficient) and f_u_ = 0.65 (fraction bound to proteins).
j) Inter-individual differences in metabolism and excretion are not explicitly considered (only deterministic modelling). To partially account for such variations, the metabolic rates are corrected by the subject’s body weight.

The mathematical equations were programmed in R language by combining functionalities of the following R packages available from “The Comprehensive R Archive Network” website (http://cran.r-project.org): deSolve, ReacTran, PK, FME, rgenoud and AICcmodavg. Ordinary differential equations (ODEs) were solved by the method *lsoda* available in the deSolve package. The method of lines was used to solve partial differential equations (PDEs). Further details of the mathematical equations are given in Appendix A1 in Supplementary material.

### 2.6 PBK model parameters

Anatomical/physiological parameters for rat and humans (reference woman and reference man) were taken from literature (Brown et al., 1997) and listed in the Appendix A2 in table A1 in Supplementary material. All physiological parameters for a reference man, woman and rat that are independent of the chemical and constitute a constant part of the model equations (Appendix A1 in Supplementary material). The ADME parameters for oral and dermal absorption of caffeine are given in Appendix A2, Table A2 Supplementary material. For the oral human model, the most sensitive parameters (GIT dissolution rates, first order uptake rate constants and metabolism parameters for liver) were optimized using measured human data. Liver metabolism parameters were taken from the literature and were optimized with respect to *in vivo* blood concentrations using human data for the main metabolites (Gajewska et al., 2015).

### 2.7 Human *in vivo* data

As reported in Gajewska et al. (2015) *in vivo* plasma concentrations of caffeine following oral absorption were taken from: (i) Lelo et al. (1986) where a non-smoking male volunteer ingested only once 270 mg of caffeine in a gelatin capsule followed by 150 mL of water; (ii) Csajka et al. (2005) where caffeine was given orally in a gelatin capsule (200 mg of caffeine sulphate) to 16 subjects and in a commercial dietary supplement (a mixture containing 200 mg caffeine and 20 mg ephedrine alkaloids) to 8 subjects. For model validation, plasma concentrations from the oral study by Newton et al. (1981) were selected, in which a gelatin capsule containing 300 mg of caffeine was administered to one male subject. Plasma caffeine levels after dermal absorption were taken from Otberg et al. (2008). In this experiment caffeine in an ethanol/propylene glycol vehicle was administered to 6 male volunteers by applying the liquid onto a chest area of 25 cm^2^ for 24 h. In contrast to other dermal absorption studies, the additional impact of hair follicles in the overall absorption process was considered.

### 2.8 Sensitivity analysis

We analyzed several parameters using the sensitivity analysis as reported in Gajewska et al. (2014). Briefly, the sensitivity analysis and parameter identifiability were performed to identify the most important and sensitive parameters with respect to the blood/plasma AUC for caffeine according to Soetaert and Petzoldt (Soetaert and Petzoldt, 2010) prior to their optimization. In local sensitivity analysis Eq. (1), all parameters were evaluated individually in a very small region close to their nominal value. A parameter value divided by the average of simulated outputs was used as a scaling factor (SF).

(1) $$\text{x}=\text{f}\left(\text{x},\text{u},\phi \right)\text{y}=\text{g}\left(\text{x},\phi \right){\text{S}}_{\text{ij}}=\left(\frac{\phi_{\text{j}0}}{\text{SF}}\right)\frac{\partial {\text{y}}_{\text{i}}}{\partial \phi_{\text{j}}}|\phi =\phi_{0}$$

(2) $$\text{L}1=\frac{\sum \left|{\text{S}}_{\text{ij}}\right|}{\text{N}}\text{L}2=\sqrt{\frac{\sum {\text{S}}_{\text{ij}}^{2}}{\text{N}}}$$

(3) $$\gamma =\frac{1}{\sqrt{\min\left(\text{EV}\right)\left[\hat{{\text{s}}^{\text{T}}}\cdot \hat{\text{s}}\right]}}$$

where y is vector of function outputs for a specific variable; x is vector of state variables; θ is vector of parameters (θ 0parameter estimate); u is vector of inputs; N is number of time points;$\hat{\text{s}}$ is the columns of the sensitivity matrix that correspond to the parameters included in the set; EV is estimation of the eigenvalues.

The following kinetic and compound-specific parameters were analyzed: GItract absorption rates (D_iss_ (or D_t_), k_astm_, k_aSI_, k_aLI_, k_elLI_, k_max_, k_min_), liver metabolic rates (V_max_, kK_m_, K_met_), skin absorption parameters (D_sc_, D_ve_, D_hf_, k_aform_, k_ahf_, PC_sc_,PC_scve_, PC_hf_), blood-to-plasma ratio and tissue-to-blood partition coefficients: PC_liv_, PC_adp_, PC_ppt_, PC_hpt_, PC_kid_, PC_lng_, PC_git_, PC_skn_. The higher the absolute sensitivity value, the more important is the parameter. These sensitivity functions are collapsed into summary values (L1 and L2 are used as selection criteria) (Eq. (2)). Based on the sensitivity functions of blood AUC to selected parameters, the identifiability of a set of parameters to be fine-tuned by calibration is then calculated. As a rule of thumb, a collinearity value (y) less than about 20 means “identifiable” (in general, when the collinearity index exceeds 20, the linear dependence is assumed to be critical (Eq. (3)) (Brun et al., 2001). The collinearity is a measure of approximate linear dependence between sets of parameters. The higher its value, the more the parameters are related. In this context, “related” means that several parameter combinations may produce similar values of the output variables.

Monte Carlo simulations were used to quantify the impact of variability and uncertainty in parameter distributions separately by drawing parameter values according to a predefined distribution (normally distributed random samples), running the model with each of these parameter combinations, and calculating the values of the selected output variables at each output interval. The parameters were optimized according to the Levenberg-Marquardt algorithm for nonlinear data fitting (Moré, 1978).

With an oral exposure to caffeine, the tissue-to-blood partition coefficients show comparable sensitivity to kinetic parameters in terms of blood/plasma AUC; however, lower sensitivity is observed in the case of dermal absorption. The most sensitive kinetic parameters are caffeine metabolic rate to paraxanthine (V_max_), skin absorption rates (D_sc_, D_ve_, D_hf_) and blood-to-plasma ratio. The most sensitive partition coefficients are: PC_adp_, PC_ppt_, PC_kid_ (oral model) and PC_lng_, PC_adp_, PC_ppt_ (dermal model).

### 2.9 QSAR predictions of skin penetration and metabolism for caffeine

The equations of the QSARs applied for assessing dermal bioavailability are given in Gajewska et al. (2014). A review of *in vitro* and QSAR methods for predicting dermal absorption is given in Dumont et al. (2015). In order to explore models for predicting liver and skin metabolism the OECD QSAR Toolbox was applied. For caffeine, five metabolites were predicted by a QSAR approach for liver metabolism and one for skin metabolism. However, in case of skin metabolism, neither *in vitro* nor *in vivo* data were found with respect to the relevance, quantity and/or the metabolic rate constants (V_max_, K_m_). Therefore, the formation of these metabolites was not included in the PBK model.

## 3 Results

### 3.1 PBK model simulations: rat study- oral dosing at NOAEL

Estimates for internal concentrations of rat venous blood and liver following oral dosing (10.1 mg/kg bw) are provided in Table 3 for caffeine and its main metabolite. Results are expressed in terms of area under the curve (AUC) and peak concentration (C_max_).

**Table 3:** Simulated systemic exposure dose metrics in rat and human.

Rat/oral	AUC liver mg h L^−1^	AUC blood mg h L^−1^	C_max_ liver mg L^−1^	C_max_ blood mg L^−1^
Caffeine	245.3	4.8	123.1	1.9
Paraxanthine	15.3	1.2	3.1	0.18

Dermal	single	multiple	single	multiple	single	multiple	single	multiple
Caffeine	65.7	472.2	19.1	127.8	2.4	7.5	0.7	1.99
Paraxanthine	39.5	168.6	10.5	44.7	1.2	2.26	0.32	0.6

### 3.2 PBK model simulations: human oral exposure at rat POD levels

**Fig. 5:**
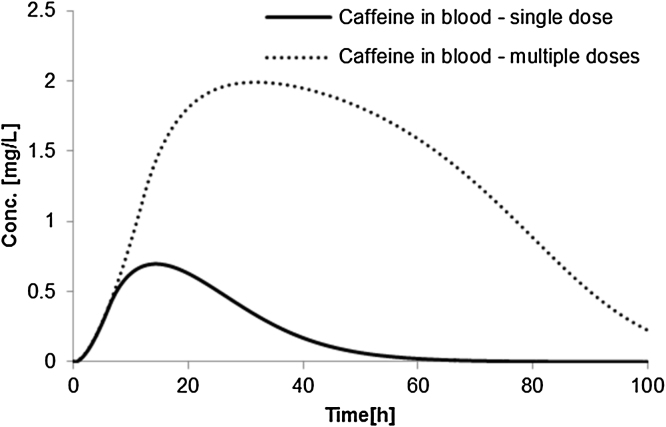
Internal (blood) concentrations of caffeine in humans following dermal exposure of 2.5 mg/kg bw (single and multiple doses).

Fig. 5 shows concentration-time profiles for caffeine in blood after single and repeated dermal exposure and Table 3 contains AUC- and C_max_-based toxicokinetic results for caffeine and its main metabolite paraxanthine. The dermal absorption of caffeine in humans is assumed to follow an extreme condition where hair follicles contribute to the overall absorption rate. In this way, absorption of caffeine in a single dose is around 86%. Although caffeine is typically applied in cream formulations, the vehicle considered here is ethanol with propylene glycol. In the repeated application scenario, skin application is done 4 times at 0, 2, 4, 6 h with a new formulation replacing the old one.

To obtain the default relative skin absorption (%) of the SCCS Notes for Guidance (2016), the water partition coefficient of the *stratum corneum* was modified (here from PCsc = 1.6 to PCsc = 1 to reach 50% of absorbed single dose).

### 3.3 Calculation of margin of internal exposure (MOIE)

The conventional MOE (Margin of Exposure) based on direct comparison of the human dermal exposure dose to the animal POD (Point of Departure) is shown in the last row in Table 2. Subsequently, Margins of Internal Exposure (MOIE based on predicted AUC or C_max_) were calculated for realistic human dermal exposure scenarios and were compared to those at rat PODs (NOAEL): MOIE = SPOD/SED based on predicted AUC or C_max_ values. The results are shown in Table 4. The MOIE was first calculated assuming that the parent chemical (caffeine) is the toxic moiety (parent chemical mode of action) and then assuming that the major metabolite (paraxanthine) formed is the toxic moiety. In addition, the MOIE was calculated assuming a single dermal exposure as well as assuming multiple (4×) exposures. The AUC and C_max_ values are shown in Table 3.

**Table 4:** Caffeine − Margins for Internal Exposure (MOIE) calculated for the dermal human exposure scenarios in comparison to a single oral dose to rat at the NOAEL and LOAEL.

DERMAL (POD Rat NOAEL 10.1 mg/kg bw/d)				
Caffeine single exposure	DERMAL 2.5 mg/kg bw/d			
	**AUC liver**	**AUC blood**	**C_max_liver**	**C_max_blood**
POD − NOAEL one day	245.3	4.8	123.1	1.9
SED	65.7	19.1	2.4	0,7
MOIE	**3.77**	**0.25**	**50.64**	**2.68**

PARAXANTHINE single exposure	DERMAL 2.5 mg/kg bw/d			
	**AUC liver**	**AUC blood**	**C_max_liver**	**C_max_ blood**
POD − NOAEL one day	15.3	1.2	3.1	0.18
SED	39.5	10.5	1.200	0.32
MOIE	0.39	0.11	2.59	0.58

Caffeine multiple exposure	DERMAL 2.5 mg/kg bw/d			
	**AUC liver**	**AUC blood**	**C_max_ liver**	**C_max_ blood**
POD − NOAEL one day	245.3	4.8	123.1	1.9
SED	472.2	127.8	7.51	1.99
MOIE	**0.52**	**0.04**	**16.40**	**0.94**

PARAXANTHINE multiple exposure	DERMAL 2.5 mg/kg bw/d			
	**AUC liver**	**AUC blood**	**C_max_ liver**	**C_max_ blood**
POD − NOAEL one day	15.3	1.2	3.1	0.18
SED	168,6	44.7	2.26	0.6
MOIE	0.09	0.03	1.37	**0.31**

Caffeine exposure scenarios were simulated and calculated to go with an acceptable (around 50) to very low (below 1) MOIE values. It is noted that the figures are purely hypothetical and used for illustrative purposes and therefore should not be used as such for direct risk assessment purposes. Further scrutiny of the usefulness of the MOIE approach based on PBK modelling is needed to decide on its practical value as a higher tier approach over the approach were only external doses and exposures are compared (MOE) or were merely default oral and dermal absorption figures are used.

## 4 Discussion

In the present work we introduce the MOIE concept based on PBK simulations of an experimental animal dosing scenario and hypothesised human dermal exposure scenarios. The developed an integrated modelling approach (PBK and QSAR modelling) as an alternative to the establishment of human dermal threshold values for caffeine as well as to lower tier risk assessment comparing only external doses and exposures (MOE). The integrated modelling approach predicts the internal dose metrics (AUC and C_max_) for caffeine that result from the oral dosing of rats, from oral dosing of volunteers (used for calibration purposes) and from human dermal exposure. The approach is technically feasible and can scrutinise the consequences of a large number of possible dermal exposure scenarios on the internal dose metrics. However, for further use in actual risk assessment, further calibration of human QSAR-integrated PBK models is warranted.

The rat model for caffeine was set up to mimic one-day oral exposure (via food) at the NOAEL value found for repeated oral dosing. The human model was set up to accommodate typical/realistic single and repeated oral exposure (gavage as was actually used in the volunteer study for the gelatin capsule containing caffeine that was administered). The human volunteer data were only used for proper calibration mainly of the elimination parameters in the PBK model. Subsequently the oral human model was extended with dermal compartments, parameterised using QSAR predictions for dermal absorption. The resulting dermal human model was calibrated using data from a dermal human volunteer study (Otberg et al., 2008). Finally, various dermal human exposure scenarios were simulated using the calibrated PBK model.

In Table 2, a summary is provided about the exposure dose levels used as well as the resulting usual MOE value, i.e. 4. It is clear that the MOE for caffeine it is already very small compared to the usual minimal value of 100. It is noted that no in depth analysis was performed on the hazard characterisation data for caffeine. The lowest value for the NOAEL for caffeine as found in the literature (10.1 mg/kg bw) was taken for granted and not further scrutinised. The MOE is defined as the ratio between the *external* dose in the animal study (in this case the NOAEL dose) and the human scenario specific *external* exposure (both in mg/kg bw). The traditional MOE approach does not take into account physiological animal to human differences that influence toxicokinetics or only (mostly default) differences in human dermal and animal oral absorption. Moreover, the MOE approach does not accommodate differences in plasma concentration-time curves caused by various exposure scenarios. A recent paper (Bessems and Geraets, 2013) illustrates the consequences of not taking these aspects, such as species- and route-specific differences in absorption into account.

When comparing the general MOE value (4) in Table 2 to the results in Table 4, the latter being MOIE values (margin of internal exposure) that are based on comparison (ratio) of the *internal* exposure dose metrics (AUC or C_max_), we can see the following. For caffeine, MOIE values for human dermal exposure can vary significantly depending on the assumptions with respect to the frequency (single or multiple), the MOA relevant chemical moiety (parent caffeine or metabolite paraxanthine), the MOA relevant dose metric as such (AUC or C_max_) and the MOA relevant target tissue/compartment (liver or blood). Some of MOIE values are higher than (Table 2), while most of them are (much) smaller than 4. So in the case of caffeine, if this would be a concrete risk assessment, it appears that the classical approach actually would not seem to be conservative at all. Incorporating the scientifically better justified (risk assessment) methodology of comparing internal human and animal exposure levels (use of data instead of default assessment factors) can result in significantly smaller margins. It is noted that in the alternative approach used by the EU SCCS (SCCS, 2016), a MOIE of 25 would be regarded as minimally required (see also Fig. 1): AF of 2.5 for interspecies differences in toxicodynamics * AF of 10 (3.2 * 3.2) for human variability (based on the fact that toxicokinetic differences are incorporated by taking into account measured or default absorption percentages (human dermal versus animal oral). There are only a few MOIE values in that order of magnitude or higher, i.e. when assuming that caffeine itself is the toxic moiety and C_max_ in liver (not blood) is the relevant dose metric. All the other MOIE values are significantly smaller than 25 or are even smaller than 1.

Based on the results presented, it is clear that various assumptions have major consequences that are quite difficult to qualify at the moment due to several uncertainties regarding the MOA (target organ as well as parent or metabolite) and dose metric (AUC or C_max_). Few assumptions were listed in Table 5 for which the quantitative consequences were not investigated further.

**Table 5:** Some assumptions, with the inherent uncertainties and possible consequences.

Assumption	Uncertainty	Potential consequence for internal dose metrics	Potential risk assessment consequences
First order oral absorption	First order oral absorption has been stated to be applicable to many pharmaceuticals. However, for non-pharmaceuticals, this is uncertain. Also, the value for the first order absorption rate constant used may change for significantly different exposure conditions.	Predicted AUC as well as C_max_values may deviate significantly from the real AUC and C_max_(rat, human).	Significant over- or underestimation of actual risk.
No human first pass metabolism in skin	First pass metabolism reduces the internal exposure to the parent chemical.	Internal exposure to the parent chemical is overestimated (either as AUC or as C_max_).	No consequence in comparison to default approach (just using total external dose). Larger risks predicted compared to when first pass metabolism in skin would have been taken into account.
No genetic polymorphism in the human population	The values used for V_max_ and K_m_ may are not valid for whole human population (it is known that there is genetic polymorphism)	Predicted AUC and V_max_ are not valid for whole human population.	Risks maybe over- or under predicted for part of the human population.

From a methodological point of view in human dermal risk assessment it is shown that interspecies differences in toxicokinetics being inherently linked to route-to-route extrapolation of rat oral toxicity data can be taken into account. It was done by integrated PBK modelling of whole organism rat and human route-dependent kinetics using QSAR predictions for human dermal penetration and assuming first order absorption kinetics using the calibration approach.

Next to various assumptions mentioned earlier, there is the issue of route-specific metabolism. Caffeine is metabolized in the liver by CYP1A2. Skin expresses little amount of CYP1A2 but CYP1A1 and CY1B1 are inducible by UV radiations and CYP1A2 by polycyclic aromatic hydrocarbons (Smith et al., 2003). For any further work, investigations on metabolism using viable excised skin or a reconstructed skin model could be used to check the assumption that first-pass metabolism in skin is not relevant or maybe come up with some more quantitative assessment of the relative contribution to the total metabolic clearance. However, in general, skin metabolism is regarded not to be an important source of metabolic clearance for most chemicals. Moreover, the overall MOIE value predicted would nevertheless still be dependent on other uncertainties.

Interestingly, the results of the integrated PBK modelling approach as shown for caffeine make it clear that simple derivation of external dermal threshold values (in mg/kg bw/d) is not trivial, and scientifically justifiability is disputable. Any external dermal threshold value derived from an animal oral toxicity study would depend very much on the actual human dermal exposure scenario used to derive it and consequently, be valid only for that use scenario. With respect to trace chemicals that might be present as food packaging migrants oral dosing scenarios in experimental animals are quite similar to actual human oral exposure. The frequency of exposure more or less follows the pattern of consuming food which is rather similar to the pattern of food consumption in experimental animals. Also, the routes of exposure are generally the same in the animal bioassay and consumers and assuming comparable absorption kinetics in the gastrointestinal tract of experimental animals and food-consuming humans is probably often a valid assumption. However, dermal exposure scenarios for cosmetic ingredients that are probably present at significantly higher concentrations in the cosmetic product than food packaging migrants in food may differ to a much wider extent. Furthermore, cosmetic product ingredients are often present in many different cosmetic products with widely varying use scenarios.

Differences between human dermal exposure scenarios can have significant effects on the qualitative aspects of the concentration-time curve (the form) as well as on the more quantitative aspects of the curve, expressed as AUC and C_max_. Multiple exposures on a single day (e.g. four times as might be the case e.g. for sun screen) may result in significantly higher (cumulating) internal exposure compared to one single external exposure. This means that using the “*reverse dosimetry*” approach, i.e. to predict human dermal exposure levels that result in an AUC and C_max_ that is equivalent to an AUC or C_max_ at the oral rat internal NOAEL divided by 100 (overall assessment factor), is not practical as there is simply not one generally applicable human use scenario for a specific chemical ingredient used in various cosmetic products. The external factors that drive the systemic exposure and that are input parameters for any PBK model (frequency of use, concentration of the chemical per cm^2^ etc.) vary that widely that it is questionable whether incorporation in a probabilistic reverse dosimetry approach would provide any useful outcome, with likely extremely wide distributions of external ‘safe’ levels). Subsequently, the question arises how cosmetics industry should translate this into ‘maximally allowed chemical concentrations in a product’.

Lastly, even if all the issues with respect to the MOA would be solved, the only way in which formulation of a dermal threshold exposure might be feasible is to assume one generally applicable human dermal exposure scenario in which many potential variables are kept constant. Examples of these variables are the concentration of the chemical in the cosmetic product, the constitution of the cosmetic product (emulsion, cream, soap, etc.), the exposure time, the frequency of exposure, the skin area exposed and parameters that are (partially) dependent on the former, such as the dermal load (SSAD = mass of chemical per cm^2^), the dermal exposure rate (how much chemical is released from the cosmetic product per cm^2^ per unit time). This would probably only make sense if a cosmetic ingredient would be used in a few products were generalisation into one exposure scenario would be acceptable. In fact, this is very often not the case.

This work is intended to illustrate how different modelling approaches can in principle be used in a risk assessment approach for cosmetic substances. The examples were chosen solely for illustrative purposes and are not intended to constitute or replace a risk assessment or expert opinion on the case study chemicals. A similar shift in the application and use of internal dose metrics in the risk assessment of chemicals passing the dermal barrier was previously illustrated and supported by Dancik et al. (2013). There work that also included PBK models highlighted the importance of a new approach to incorporate the impact of dermal exposure scenarios and application conditions in risk assessment. It also underpinned the importance that internal dose metrics will have in establishing also a better dose response relationship, i.e. an internal exposure dose response relationship. Previously, in the RCR approach (Fig. 1 on the left), the human equivalent oral dose to the rat oral NOAEL was established by simple allometric scaling (Gajewska et al., 2014). In the current paper both rat blood and liver dose metrics as well as human blood and liver dose metrics were predicted by PBK modelling in the MOIE approach (Fig. 1 on the right). Finally, in order to improve the actual dermal load kinetics linked to use scenarios of specific cosmetic products, the MOIE approach could be integrated with consumer exposure models such as ConsExpo as developed by RIVM (http://www.rivm.nl/en/Topics/C/ConsExpo). ConsExpo enables the estimation and assessment of exposure doses to chemical ingredients in consumer products such as paint, cleaning agents but also personal care products.

## 5 Conclusions

In summary, the current paper, presents for the first time an approach that is defined as the Margin of Internal Exposure (MOIE) approach. It could in principle make risk assessment more transparent to the risk manager. It offers opportunities to adapt initial parameter values in future with better data, i.e. by improved reliability of a QSAR used or simply by relevant *in vitro* measurement e.g. of dermal absorption or metabolism. Furthermore, it illustrates the importance of a proper mode of action hypothesis and can accommodate changing insights in a transparent way: for instance, is the parent chemical or a metabolite (more) relevant in the proposed mode of action and what is the target organ and is the AUC or the C_max_ more relevant? This offers excellent opportunities for the animal-free approach as imposed by the EU ban on animal testing for cosmetic products and ingredients (EU Regulation 1223/2009).

The MOIE approach can be applied to other organ/s or tissue/s once new information indicates that following human dermal exposure the liver might not be the most sensitive/target organ and if no clarity exists to take the best proxy (blood). It can also be adapted to include human *in vitro* cellular systems dose metrics (intracellular or cell medium) in a time-frame of integrated risk assessment, decreasing the dependence on animal bioassays derived dose metrics. This new approach clearly provides transparency advantages; additionally, it provides opportunities to add on information with respect to data uncertainty as well as human variability. It even might improve risk assessment by incorporating more human based ADME and TK data/values including experimental data generated by *in vitro* or *in silico* methods as input variables in PBK models (Bessems et al., 2015). Finally, the great advantage of applying the MOIE approach is that different exposure scenarios, as in the case for cosmetics ingredients, can be assessed for human health risk.

## Footnotes

1 The term ‘kinetic’ can be read as such or as ‘pharmacokinetic’, ‘toxicokinetic’ or ‘biokinetic’. The acronyms linked to those terms are PBK, PBPK, PBTK and PBBK, respectively.
