# Supplementary material for: The margin of internal exposure (MOIE) concept for dermal risk assessment based on oral toxicity data – A case study with caffeine
Source: Toxicology. 2017 Dec 1;392:119–29. doi: 10.1016/j.tox.2017.03.012 (PMC5699174; doi:10.1016/j.tox.2017.03.012)
Supplement: Supplementary file 1 [file mmc1.docx]

**Appendices**

**Journal**: Toxicology

As *Original research Article*

**Title**: The margin of internal exposure (MOIE) concept for dermal risk assessment based on oral toxicity data – a case study with caffeine

**Authors**: Jos GM Bessems^a^, Alicia Paini^a,^ *, Monika Gajewska^a^, Andrew Worth^a,^

**Affiliation**:

^a^ Directorate Health, Consumers and Reference Materials, European Commission, Joint Research Centre, Ispra, Italy

(*) Corresponding author: alicia.paini@ec.europa.eu

Health, Consumers and Reference Materials Directorate

Chemical Safety and Alternative Methods Unit

Via E. Fermi 2749, TP 126 21027 Ispra (VA), Italy

Via E. Fermi 2749, TP 126

I-21027 Ispra (VA), Italy

tel.+39-0332-78 3986

fax +39-0332-78 9963

# Appendix 1 - Mathematical equations for the PBK models (Gajewska et al., 2014; 2015)

- Adipose tissue and highly- and poorly-perfused tissues

$\frac{dA_{org}}{dt}=f_{org}\cdot\left(C_{art}-\frac{C_{org}}{PC_{org}}\right)\text{ , }A_{org}(t=0)=0\text{ , }C_{art}=\frac{C_{art}}{V_{art}}\text{ }C_{org}=\frac{A_{org}}{V_{org}}$ (A1.1)

where: org = organ name (adp, hpt, ppt)

- Metabolism is assumed to occur mainly in the liver

 (A1.2)

Where, for a parent compound:

$$Human:Fl_{GIT}=fra\cdot f_{git}\cdot\frac{C_{stm}}{PC_{git}}+frb\cdot f_{git}\cdot\frac{C_{SI}}{PC_{git}}+frc\cdot f_{git}\cdot\frac{C_{LI}}{PC_{git}}$$

$$Rat:Fl_{GIT}=ka_{GI,rat}\cdot\frac{C_{GI,rat}}{PC_{GI,rat}}$$

$FORM_{liv}=0$ (rate of formation of metabolites)

In most cases, metabolism was assumed to follow Michaelis–Menten kinetics (A1.3) or was described by the first order reaction (A1.4). The liver metabolic parameter values as calibrated and optimised using the oral human volunteer data were taken unchanged to the human dermal model.

$\frac{\text{V}_{\text{max}}\cdot\frac{C_{liv}}{PC_{liv}}\text{ }}{\text{K}_{\text{m}}+\frac{C_{liv}}{PC_{liv}}}$ (A1.3)

$K_{\text{met}}\cdot\frac{C_{liv}}{PC_{liv}}$ (A1.4)

where:

C_liv_ is the liver concentration and PC_liv_ is the liver-to-blood partition coefficient of a substance undergoing metabolism.

 (A1.5)

*Venous blood mass balance is as follows:*

$\frac{dA_{ven}}{dt}=f_{liv}\cdot\frac{C_{liv}}{PC_{liv}}+f_{ppt}\cdot\frac{C_{ppt}}{PC_{ppt}}+f_{hpt}\cdot\frac{C_{hpt}}{PC_{hpt}}+f_{adp}\cdot\frac{C_{adp}}{PC_{adp}}+f_{kid}\cdot\frac{C_{kid}}{PC_{kid}}+Fl_{skn}-f_{crd}\cdot C_{ven}\text{ , }A_{ven}(t=0)=0\text{ , }C_{ven}=\frac{A_{ven}}{V_{ven}}$(A1.6)

where, for dermal absorption only: $Fl_{skn}=\frac{3}{4}\cdot f_{skn}\cdot\frac{C_{skn}}{PC_{skn}}+\frac{1}{4}\cdot f_{skn}\cdot\frac{C_{sknhf}}{PC_{skn}}$ otherwise $Fl_{skn}=0$

We assume that plasma accounts for 55% of blood volume.

Plasma quantification: $C_{ven,PL}=\frac{\frac{A_{ven}}{(0.55\cdot V_{ven})}}{RBP}$ (A1.7)

*Arterial blood mass balance:*

$\frac{dA_{\ln g}}{dt}=f_{\ln g}\cdot\left(C_{ven}-\frac{C_{\ln g}}{PC_{\ln g}}\right)\text{ ; }A_{\ln g}(t=0)=0\text{ ; }C_{\ln g}=\frac{A_{\ln g}}{V_{\ln g}}$ (A1.8)

$\frac{dA_{art}}{dt}=f_{crd}\cdot\left(\frac{C_{\ln g}}{PC_{\ln g}}-C_{art}\right)\text{ ; }A_{art}(t=0)=0$ (A1.9)

*Rat oral absorption*

A single compartment representing the GI tract with first rate of absorption:

$\frac{dA_{GI,rat}}{dt}=-ka_{GI,rat}\cdot\frac{C_{GI,rat}}{PC_{GI,rat}}$ (A1.10)

*Human GI tract model consists of sub-compartments:*

Stomach content:

$\frac{dA_{stm,cont}}{dt}=D_{rt}-ka_{stm}\cdot C_{stm,cont}-kGIT\cdot A_{stm,cont}$ (A1.11)

with: $\text{ }C_{stm,cont}=\frac{A_{stm,cont}}{V_{stm}},\text{ }kGIT=\frac{k_{\max}}{(1+k_{\min}\cdot C_{stm})}$

and:

administration via gavage: $D_{rt}=0\text{ and }A_{stm,cont}(t=0)=Dose$

administration by a coated tablet: $D_{rt}=Diss\cdot\frac{dA_{stm,cont,diss}}{dt}\text{ and }A_{stm,cont,diss}(t=0)=dose,\text{ }A_{stm,cont}(t=0)=0$

Stomach tissue:

$\frac{dA_{stm}}{dt}=ka_{stm}\cdot C_{stm,cont}+fra\cdot f_{git}\cdot\left(C_{art}-\frac{C_{stm}}{PC_{git}}\right)\text{, }A_{stm}(t=0)=0,\text{ }C_{art}=\frac{A_{art}}{V_{art}}\text{ , }C_{stm}=\frac{A_{stm}}{V_{stm}}$ (A1.12)

Small intestine lumen and absorbed quantity:

$\frac{dA_{SI,lumen}}{dt}=kGIT\cdot A_{stm,cont}-(ka_{SI}+flow_{LI})\cdot C_{SI,lumen}\text{, }A_{SI,lument}(t=0)=0\text{ , }C_{SI,lumen}=\frac{A_{SI,lumen}}{\left(\frac{3}{4}\cdot V_{\mathrm{int}}\right)}$ (A1.13) $\frac{dA_{SI}}{dt}=ka_{SI}\cdot C_{SI,lumen}+frb\cdot f_{git}\cdot\left(C_{art}-\frac{C_{SI,}}{PC_{git}}\right)\text{, }A_{SI}(t=0)=0\text{ , }C_{SI}=\frac{A_{SI}}{\left(\frac{3}{4}\cdot V_{\mathrm{int}}\right)}$ (A1.14)

Large intestine lumen and absorbed quantity:

$\frac{dA_{LI,lumen}}{dt}=flow_{LI}\cdot C_{SI,lumen}-\left(kel_{LI}+ka_{LI}\right)\cdot C_{LI,lumen}\text{, }A_{LI,lumen}(t=0)=0,\text{ }C_{LI,lumen}=\frac{A_{LI,lumen}}{\left(\frac{1}{4}\cdot V_{\mathrm{int}}\right)}$ (A1.15)

$\frac{dA_{LI}}{dt}=ka_{LI}\cdot C_{LI,lumen}+frc\cdot f_{git}\cdot\left(C_{art}-\frac{C_{LI}}{PC_{git}}\right)\text{, }A_{LI}(t=0)=0\text{ , }C_{LI}=\frac{A_{LI}}{\left(\frac{1}{4}\cdot V_{\mathrm{int}}\right)}$ (A1.16)

*Skin surface:*

$or(t<t_{appl})\text{ }AbsRate=-(ka_{form}\cdot C_{form}+ka_{hf}\cdot C_{form})\text{ and }for(t>t_{appl})\text{ }AbsRate=0$ (A1.17)

Where: t_appl_ is the application time of a formulation on the skin.

Evaporation of a vehicle (EvapRate) is calculated for volatile substances according to Tibaldi *et al.,* (Tibaldi, ten Berge, & Drolet, 2011):

$EvapRate=\frac{-k_{evp}}{TL}$ (A1.18)

For a vehicle (solvent) this evaporation is quantified in terms of decrease of an applied solution volume in time rather than mass of the solvent:

$\frac{dV_{appliedForm}}{dt}=-\frac{k_{evp}\cdot Area}{1000\cdot\sigma_{form}}\text{ }V_{appliedForm}(t=0)=V_{0,appliedForm}$ (A1.19)

where: $k_{evp}=\frac{\beta\cdot MW\cdot V_{p}}{R\cdot T\cdot10}\left[\frac{mg}{h\cdot cm^{2}}\right]\text{ }\beta=\frac{0.0111\cdot\nu_{air}^{0.96}\cdot D_{g}^{0.19}}{\upsilon^{0.15}\cdot X^{0.04}}$

where *β* is the mass transfer coefficient in vapour phase (m h^-1^), *MW* the molecular weight, *V_p_* the vapour pressure of the liquid at skin temperature (Pa), *R* the gas constant in J mol^-1^K^-1^, *T* the skin temperature (assumed to be 303K which equals 29.85°C), *V_air_* the velocity of air (at workplaces it ranges from 0.3-0.6 m s^-1^), *D_g_* the diffusivity of the liquid in gas phase (range: 0.03 to 0.06 m^2^h^-1^), ϑ the kinematic viscosity of air (literature value of 0.054 m^2^ h^-1^), *X* the length of evaporation area in the direction of air stream, *TL* a thickness of applied substance layer (cm), *Area* the application area (cm^2^) and *δ_form_* the density of solvent (g cm^-3^).

*Stratum corneum (SC):*

Passive diffusion is modelled according to Fick’s second law of unidimensional diffusion with initial and boundary conditions (t_appl_ is a time of experiment duration, after which the remaining formulation is wiped off from skin surface). The diffusion coefficient is assumed to be constant throughout the process:

 (A1.20)

where: $q_{SC.,i}=-Dsc_{i}\cdot\frac{C_{SC,i+1}-2\cdot C_{SC,i}+C_{SC.,i-1}}{\left(\frac{Lsc}{N}\right)};i=1:N$

Initial and boundary conditions:

$$\begin{matrix} C_{SC}(t=0)|_{0\leq x\leq L_{SC}}=0 \\ \frac{dC_{SC}}{dt}(t>0)|_{x=0}=ka_{form}\cdot C_{form} \\ D_{SC}\cdot\frac{dC_{SC}}{dx}(t>t_{appl})|_{x=0}=0 \\ C_{SC}(t>0)|_{x=0}=PC_{SC}\cdot C_{form} \\ C_{SC}(t>0)|_{x=L_{SC}}=PC_{SCVE}\cdot C_{VE} \end{matrix}$$

*Viable epidermis (VE):*

The reaction-diffusion partial differential equations for viable epidermis are solved in the same manner, by means of the methods of lines approach:

 (A1.21)

Where:

Initial and boundary conditions:

$$\begin{matrix} C_{VE}(t=0)|_{0\leq y\leq L_{VE}}=0 \\ C_{VE}(t>0)|_{y=0}=\frac{C_{SC,i=N}}{PC_{SCVE}} \\ D_{SC}\frac{dC_{SC}}{dx}|_{x=L_{SC}}=D_{VE}\cdot\frac{dC_{VE}}{dy}|_{y=0} \\ C_{VE}(t>0)|_{y=L_{VE}}=PC_{skn}\cdot C_{skn} \end{matrix}$$

If skin metabolism occurs, its rate is assumed to follow first order kinetics (until more data become available). The model simulations were run for N=M=10 layers.

*Dermis and mix with blood:*

$\frac{dA_{skn}}{dt}=\frac{3}{4}\cdot f_{skn}\cdot\left(C_{art}-\frac{C_{skn}}{PC_{skn}}\right)+q_{VE,j=M}\cdot\frac{100-nf}{100}\cdot Area\text{ }A_{skn}(t=0)=0\text{ }C_{skn}=\frac{A_{skn}}{10^{-3}\cdot\frac{2}{3}\cdot Vde}$ (A1.22)

*Hair follicles compartment* (Bookout Jr., Quinn, & McDougal, 1997)*:*

 (A1.23)

The initial and boundary conditions:

$$\begin{matrix} C_{hf}(t=0)|_{0\leq x\leq L_{hf}}=0 \\ \frac{dC_{hf}}{dt}(t>0)|_{x=0}=ka_{hf}\cdot C_{form} \\ D_{hf}\cdot\frac{dC_{hf}}{dx}(t>t_{appl})|_{x=0}=0 \\ C_{hf}(t>0)|_{x=0}=PC_{hf,}\cdot C_{form} \\ C_{hf}(t>0)|_{x=L_{hf}}=PC_{Skn}\cdot C_{sknhf} \end{matrix}$$

*Hair follicles: mix with blood*

$\frac{dA_{skinhf}}{dt}=\frac{1}{4}\cdot f_{skn}\cdot\left(C_{art}-\frac{C_{sknhf}}{PC_{skn}}\right)+q_{hf,i=K}\cdot\frac{nf}{100}\cdot Area\text{ }A_{sknhf}(t=0)=0\text{ }C_{sknhf}=\frac{A_{sknhf}}{10^{-3}\cdot\frac{1}{3}\cdot Vde}$ (A1.24)

**List of symbols**

| **Symbol** | **Parameter [unit]** |
| --- | --- |
| A_org_ | amount of a chemical in organ/tissue [mg] |
| Area | application area on skin [cm^2^] |
| CLR | renal clearance rate [L/h] |
| C_org_ | concentration of a chemical in organ/tissue [mg/L] |
| D_HF_ | diffusion coefficient in coefficient in hair follicles [cm^2^/h] |
| Diss | dissolution from a coated matrix [L/h] |
| D_SC_ | diffusion coefficient *in stratum corneum* [cm^2^/h] |
| Dt | drinking rate [L/h] |
| D_VE_ | diffusion coefficient in viable epidermis [cm^2^/h] |
| f_crd_ | cardiac output [L/h] |
| flow_LI_ | flow rate from small intestine into large intestine [L/h] |
| f_org_ | regional blood flow rates [L/h] |
| Fl_GIT_ | Amount from the GI tract [mg] |
| Flskn | Amount from the skin [mg] |
| ka_form_ | intake rate of chemical from formulation by stratum corneum [mL/h] |
| ka_HF_ | intake rate of a chemical from formulation by hair follicles [mL/h] |
| ka_LI_ | absorption rate from large intestine lumen [L/h] |
| ka_SI_ | absorption rate from small intestine lumen [L/h] |
| ka_stm_ | absorption rate into stomach tissue [L/h] |
| ka_GI,rat_ | absorption rate into GI tract for rat [L/h] |
| kel_LI_ | elimination rate in large intestine lumen [L/h] |
| K_m_ | chemical concentration at which the reaction rate is half of the maximal [mg/L] |
| K_met_ | first order rate of formation of metabolites [L/h] |
| K_min_, K_max_ | kinetic constants of stomach emptying rate of a chemical to small intestine [L/h] |
| L_sc_ | thickness of *stratum corneum* [cm] |
| L_ve_ | thickness of viable epidermis [cm] |
| PC_bloodair_ | blood/air partition coefficient |
| PC_HF_ | partition coefficient hair follicle /solvent |
| PC_org_ | tissue-to- blood partition coefficients |
| PC_SC_ | partition coefficient *stratum corneum*/ solvent |
| PC_SCVE_ | partition coefficient *stratum corneum*/ viable epidermis |
| RBP | Blood-to-plasma concentration ratio |
| Rem_bld_ | bladder emptying rate [L/h] |
| Rform_Creat_ | formation rate of creatinine[mg/h] |
| V_max_ | the maximum rate metabolic rate at maximum (saturating) concentration of a chemical [mg/h] |
| V_org_ | volume of organ/tissue |

# Appendix 2 – Parameters for the PBTK models

**Table A1**. Anatomical and physiological parameters for a reference woman, a reference man and rat (Brown et al., 1997)

| **Parameter** | **Reference woman** | **Reference man** | **Rat** |
| --- | --- | --- | --- |
| Average body weight [kg] | 65 | 75 | 0.265 |
| *Organ weights fractions (fractions of body weight)* | | |  |
| Liver | 0.026 | 0.026 | 0.0253 |
| Adipose tissue | 0.278 | 0.155 | 0.07 |
| Lungs | 0.0105 | 0.012 | 0.005 |
| Kidney | 0.0044 | 0.0044 | 0.0073 |
| GI tract total  Stomach  Small intestine  Large intestine | 0.0265 *^*^*  0.00337  0.0146  0.0085 | 0.025 *^*^*  0.00318  0.0138  0.0080 | 0.046 |
| Poorly perfused tissues + skin | 0.436 | 0.525 | 0.667 |
| Highly perfused tissues | 0.153 | 0.181 | 0.1254 |
| Blood total  Venous blood | 0.065  0.04875 | 0.072  0.054 | 0.054  0.018 |
| *Thickness of skin [cm]* |  |  |  |
| Whole skin | 0.204 | 0.2906 | 0.17 |
| Viable epidermis | 0.0032 | 0.0047 | 0.001 |
| *Stratum corneum* | 0.0018 | 0.0017 | 0.002 |
| Dermis | ? | ? | ? |
| *Organ and tissue blood flow rates [fraction of cardiac output [L/h]* | | |  |
| Cardiac output [L/h] | 15^.^ BW^0.74^ | | 5.208 |
| Liver | 0.25 | 0.24 | 0.009 |
| Adipose tissue | 0.055 | 0.04 | 0.07 |
| Skin | 0.05 | 0.05 | 0.058 |
| Highly perfused tissues | 0.155 | 0.155 | 0.222 |
| Poorly perfused tissues | 0.135 | 0.16 | 0.4 |
| Kidney | 0.19 | 0.2 | 0.14 |
| GI tract | 0.14 | 0.13 | 0.08 |
| Lungs | 0.025 | 0.025 | 0.021 |

^*^ Sum of stomach, small intestine and large intestine

**Table A2.** Human and Rat oral physiological parameters and ADME parameters for caffeine^1^

| **Parameter** | **Value** |
| --- | --- |
| *Physiological parameters oral* |  |
| Stomach emptying maximum rate k_max_ [L/h] | 8.16 (Loizou & Spendiff, 2004) |
| Stomach emptying rate k_min_ [L/h] | 0.005 (Loizou & Spendiff, 2004) |
|  |  |
| *Release from formulation/matrix/capsule* |  |
| Dissolution from matrix Diss [L/h] | 3.2 |
|  |  |
| *Absorption oral* |  |
| Stomach 1^st^ order absorption rate constant ka_stm_ [L/h] | 0.2 |
| Small intestine 1^st^ order absorption rate constant ka_si_ [L/h] | 1.5 |
| Large intestine elimination kel_li_ [L/h] | 0.1 |
|  |  |
| *Absorption dermal* |  |
| Diffusion coefficient in stratum corneum D_sc_ [cm^2^/h] | 1.4e-07 (Hansen et al., 2008) |
| Diffusion coefficient in viable epidermis D_ve_ [cm^2^/h] | 1.5e-05 ^2^ |
| Diffusion coefficient in hair follicles D_hf_ [cm^2^/h] | 1.243e-05 |
| Formulation intake rate from stratum corneum ka_form_ [mL/h] | 0.2 |
| Formulation intake rate from hair follicles ka_hf_ [mL/h] | 0.153 |
| Partition coefficient stratum corneum/vehicle PC_sc_ | 2.5 (Hansen et al., 2008) |
| Partition coefficient stratum corneum/viable epidermis PC_scve_ | 0.6 ^2^ |
| Partition coefficient hair follicles/vehicle PC_hf_ | 1 ^3^ |
| % of hair follicles in skin (nf) | 20 |
|  |  |
| *Blood/plasma ratio and tissue to blood partition coefficients (Schmitt, 2008)* |  |
| Blood-to-plasma ratio RBP | 0.28-0.35 (Newton et al., 1981) |
| Liver: PC_liv_ | 4.25 |
| Poorly-perfused tissues: PC_ppt_ | 0.995 |
| Highly-perfused tissues:PC_hpt_ | 1 |
| Skin: PC_skn_ | 1 |
| Lungs: PC_lng_ | 1.23 |
| Kidney: PC_kid_ | 3.76 |
| GI tract: PC_git_ | 1.49 |
| Adipose tissue: PC_adp_ | 0.68 |
|  |  |
| *Michaelis-Menten parameters* |  |
| caffeine to paraxanthine | V_max_ = 0.351^4^; K_m_= 1 |
| caffeine to theobromine | V_max_ = 0.0432; K_m_=1  (Lelo et al., 1986; Zandvliet et al., 2005) |
| caffeine to theophylline | V_max_ = 0.0072; K_m_=1  (Lelo et al., 1986; Zandvliet et al., 2005) |
| caffeine to trimethyluric acid | K_met_=0.001  (Lelo et al., 1986; Zandvliet et al., 2005) |

^1^ Values without footnote or reference were fitted to experimental data; ^2^ Estimated by QSPRs; ^3^ PC_hf_ was set at 1 as no QSARs were found to predict it. Optimising it to a calibration set did not make sense as the diffusion coefficient had to be optimised as well and the two are strongly correlated. As the model is more sensitive to diffusion coefficient it was decided to optimise only that value in the usual range of values of diffusion coefficient (10^-5^) and to set PC_hf_ for caffeine at 1, being a reasonable value between the usual boundaries of approx. 0.5 and 8 seen so far with this sort of chemicals; ^4^ Predicted by ADMET Predictor (https://www.simulations-plus.com/)

**Reference**

Bookout Jr., R.L. Quinn, D.W. McDougal J.N. (1997) Parallel dermal subcompartments for modeling chemical absorption. SAR QSAR Environ. Res., 7, pp. 259–279.

Brown, R.P., Delp, M.D Brown, R.P., Delp, M.D., Lindstedt, S.L., Rhomberg, L.R., Beliles, R.P., 1997. Physiological parameter values for physiologically based pharmacokinetic models. Toxicol. Ind. Health 13, 407–484.., Lindstedt, S.L., Rhomberg, L.R., Beliles, R.P., 1997. Physiological parameter values for physiologically based pharmacokinetic models. Toxicol. Ind. Health 13, 407–484.

Gajewska, M., Worth, A., Urani, C., Briesen, H., & Schramm, K. (2014). Application of physiologically-based toxicokinetic modelling in oral-to-dermal extrapolation of threshold doses of cosmetic ingredients. Toxicol Lett., 227(3).

Gajewska, M., Paini A., Sala Benito J, Burton J, Worth, A., Urani, C., Briesen, H., & Schramm, K. (2015). In vitro to in vivo correlation of the skin penetration, liver clearance and hepatotoxicity of caffeine. Food and Chemical Toxicology, 75, 39–49.

Hansen, S., Henning, A., Naegel, A., Heisig, M., Wittum, G., Neumann, D., … Schaefer, U. F. (2008). In-silico model of skin penetration based on experimentally determined input parameters. Part I: Experimental determination of partition and diffusion coefficients. *European Journal of Pharmaceutics and Biopharmaceutics*, *68*(2), 352–367.

Lelo, A., Birkett, D. J., Robson, R. A., & Miners, J. O. (1986). Comparative pharmacokinetics of caffeine and its primary demethylated metabolites paraxanthine, theobromine and theophylline in man. *British Journal of Clinical Pharmacology*, *22*(2), 177–182. Retrieved from http://www.scopus.com/inward/record.url?eid=2-s2.0-0023025836&partnerID=40&md5=aff5345dfec1380a75a9781e56072faf

Loizou, G. D., & Spendiff, M. (2004). A human PBPK model for ethanol describing inhibition of gastric motility. *Journal of Molecular Histology*, *35*(7), 687–696.

Newton, R., Broughton, L. J., Lind, M. J., Morrison, P. J., Rogers, H. J., & Bradbrook, I. D. (1981). Plasma and salivary pharmacokinetics of caffeine in man. *European Journal of Clinical Pharmacology*, *21*(1), 45–52.

Tibaldi, R. ., ten Berge, W., & Drolet, D. (2011). *IH SkinPerm Help Manual*.

Zandvliet, A. S., Huitema, A. D. R., De Jonge, M. E., Den Hoed, R., Sparidans, R. W., Hendriks, V. M., … Beijnen, J. H. (2005). Population pharmacokinetics of caffeine and its metabolites theobromine, paraxanthine and theophylline after inhalation in combination with diacetylmorphine. *Basic and Clinical Pharmacology and Toxicology*, *96*(1), 71–79. Retrieved from http://www.scopus.com/inward/record.url?eid=2-s2.0-13844310313&partnerID=40&md5=b27d1606e33e9ae71c458ac3421c660f
